# BM-BronchoLC - A rich bronchoscopy dataset for anatomical landmarks and lung cancer lesion recognition

**DOI:** 10.1038/s41597-024-03145-y

**Published:** 2024-03-28

**Authors:** Van Giap Vu, Anh Duc Hoang, Thu Phuong Phan, Ngoc Du Nguyen, Thanh Thuy Nguyen, Duc Nghia Nguyen, Ngoc Phu Dao, Thi Phuong Lan Doan, Thi Thanh Huyen Nguyen, Thi Huong Trinh, Thi Le Quyen Pham, Thi Thu Trang Le, Phan Thi Hanh, Van Tuyen Pham, Van Chuong Tran, Dang Luu Vu, Van Luong Tran, Thi Thu Thao Nguyen, Cam Phuong Pham, Gia Linh Pham, Son Ba Luong, Trung-Dung Pham, Duy-Phuc Nguyen, Thi Kieu Anh Truong, Quang Minh Nguyen, Truong-Thuy Tran, Tran Binh Dang, Viet-Cuong Ta, Quoc Long Tran, Duc-Trong Le, Le Sy Vinh

**Affiliations:** 1 Bach Mai hospital, Hanoi, 10000 Vietnam; 2 Hanoi Medical University, Hanoi, 10000 Vietnam; 3 University of Engineering and Technology, Vietnam National University, Hanoi, 10000 Vietnam

**Keywords:** Diagnosis, Medical imaging

## Abstract

Flexible bronchoscopy has revolutionized respiratory disease diagnosis. It offers direct visualization and detection of airway abnormalities, including lung cancer lesions. Accurate identification of airway lesions during flexible bronchoscopy plays an important role in the lung cancer diagnosis. The application of artificial intelligence (AI) aims to support physicians in recognizing anatomical landmarks and lung cancer lesions within bronchoscopic imagery. This work described the development of BM-BronchoLC, a rich bronchoscopy dataset encompassing 106 lung cancer and 102 non-lung cancer patients. The dataset incorporates detailed localization and categorical annotations for both anatomical landmarks and lesions, meticulously conducted by senior doctors at Bach Mai Hospital, Vietnam. To assess the dataset’s quality, we evaluate two prevalent AI backbone models, namely UNet++ and ESFPNet, on the image segmentation and classification tasks with single-task and multi-task learning paradigms. We present BM-BronchoLC as a reference dataset in developing AI models to assist diagnostic accuracy for anatomical landmarks and lung cancer lesions in bronchoscopy data.

## Background & Summary

Pioneered by Dr. Shigeto Ikeda^1^, flexible bronchoscopy has revolutionized the diagnosis and treatment of respiratory diseases. It has emerged as a crucial treatment recommended for numerous respiratory illnesses^2^. Flexible bronchoscopy enables direct visualization and identification of airway lesions by utilizing a fiber-optic light source located at the distal end of the scope, hence it allows to access the lesions and specimen collection for histopathological examination. Generally, flexible bronchoscopy is an effective procedure with a low reported rate of complications (1.08%) and fatalities (0.02%)^3^.

Flexible bronchoscopy is an indispensable diagnostic tool for diagnosing lung cancer, a malignancy with a notably high fatality rate, responsible for 18% of all cancer-induced mortalities^4^. The utilization of flexible bronchoscopy has become widespread globally throughout the past two decades^5–7^. The sensitivity of flexible bronchoscopy in detecting lung cancer was reported at 88% for central tumors and 78% for peripheral ones^8^. Accurate detection of airway lesions during flexible bronchoscopy plays a pivotal role in the lung cancer diagnosis process. Nevertheless, the effectiveness of this procedure is limited due to the reliance on subjective assessments made by the endoscopists^9^.

The integration of artificial intelligence (AI) models in augmenting lung cancer diagnosis via chest X-ray and CT scans has begun in clinical settings. Contemporary research underscored substantial benefits that accrue by the combination of bronchoscopy with deep learning technologies, improving the diagnosis and assessment of lung cancer. The core strategy involves deploying sophisticated machine learning algorithms to assist the interpretation of bronchoscopic images^10^. In terms of diagnostic accuracy, convolutional neural networks have been employed to get remarkable performance for medical image analysis systems. The adoption of pre-trained Mix Transformers is gaining traction, offering real-time lesion segmentation with promising metrics such as Intersection over Union (IoU) indices and high inference frame rate^11^. Additionally, leveraging image recognition technologies in bronchoscopic diagnostics has yielded satisfactory outcomes in terms of accuracy, sensitivity, specificity, and area under the curve (AUC) metrics^12^. These applications confirm the immense potential of integrating bronchoscopy with deep learning to enhance the precision and efficacy of lung cancer diagnosis and treatment planning. Nevertheless, further research is imperative to fully explore these encouraging advancements and their broader therapeutic implications in this swiftly growing domain.

To enhance the accurate detection of airway lesions during flexible bronchoscopy for lung cancer diagnosis, we have developed a specialized bronchoscopy dataset named BM-BronchoLC. This dataset was derived from flexible bronchoscopy images of 106 lung-cancer and 102 non-lung cancer patients. Senior bronchoscopists at Bach Mai Hospital in Vietnam meticulously annotated these images, marking both anatomical landmarks and airway lesions. To the best of our knowledge, BM-BronchoLC is the first bronchoscopy dataset which comprises rich information on the precise localization and identification of anatomical landmarks and airway lesions. To assess the dataset’s quality, we conducted experiments utilizing two prominent AI backbone models, namely UNet++ and ESFPNet, for image segmentation and classification under both single-task and multi-task learning paradigms. Preliminary findings indicate that BM-BronchoLC exhibits substantial potential as a benchmark dataset for the advancement of AI models, helping improve diagnostic accuracy for the identification of anatomical landmarks and lung cancer lesions.

## Methods

This research utilized flexible bronchoscopy videos from 208 patients, all above the age of 18, who received diagnosis and treatment at Bach Mai Hospital. Being a retrospective study that did not impact the treatment of these patients, the hospital’s ethics board granted approval for the data collection, annotation, and dissemination, waiving the need for patient consent (the approval number: 1139/BM - HĐĐĐ). To safeguard patient privacy, all identifiable personal information was manually obscured using blurred boxes before making the retrospective data publicly accessible.

**Fig. 1:**
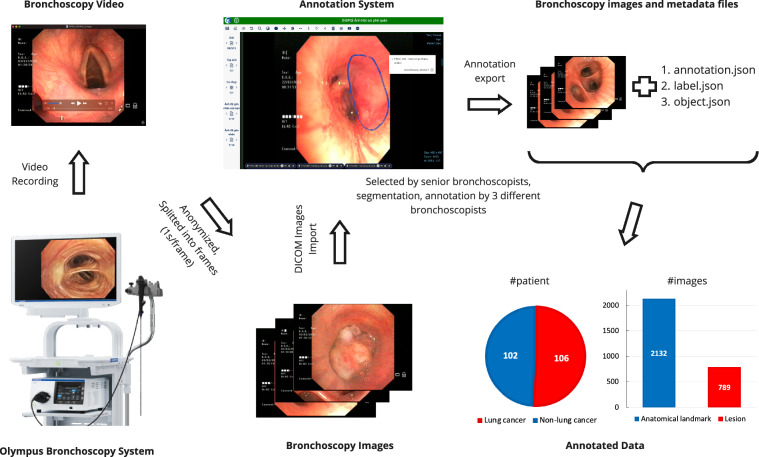
General workflow to produce the BM-BronchoLC dataset.

Figure 1 illustrates the construction workflow of the BM-BronchoLC dataset. The Olympus bronchoscopy system used the diagnostic bronchoscope to record a total of 208 bronchoscopy videos of 106 lung cancer patients and 102 non-lung cancer individuals, which served as the foundation for this retrospective dataset. To address privacy concerns, these videos were anonymized by removing all patient-sensitive information. Subsequently, the videos were segmented into frames at a rate of one frame per second. Each case study’s frames were uploaded to a specialized annotation system hosted on a secure private server. Within this system, senior bronchoscopists were tasked with selecting a minimum of ten high-quality images per case (as detailed in the “*video frame selection*” section). After the selection process, three other bronchoscopists carried out segmentation and classification tasks on these images. The annotated images, along with the respective metadata files, were then exported. The metadata files included *label.json* for landmark and lesion tags, *object.json* for object identification via bounding boxes and *annotation.json* for segment description. The final dataset comprised a set of 2,132 images depicting anatomical landmarks and 789 images for lesions, collectively representing data from 208 patients.

### Image protocol

The flexible bronchoscopy procedures in this study were conducted by respiratory specialists at the Respiratory Center of Bach Mai Hospital. These procedures were performed under either local anesthesia or intravenous anesthesia. The bronchoscope manufactured by Olympus has a working length of 600 mm, a diameter ranging from 4 to 6 mm, a direction view of 0 degree, a field view of 120 degrees, and a depth of field extending from 3 to 100 mm. Patients undergoing bronchoscopy while in the supine position, with the bronchoscope inserted via either the nose or mouth. During the procedure, bronchoscopists sequentially observed the structures of the lower respiratory tract including the vocal cords, trachea, main bronchus, lobar bronchus, and segmental bronchus. Once airway lesions were identified, tissue specimens were collected for histopathological examination. Each patient’s bronchoscopic video was stored in the*.mpeg* format on a private server at Bach Mai Hospital.

The flexible bronchoscopy videos were randomly selected from a collection spanning from 2020 to 2023. To avoid selection bias in the development of AI models for localization of anatomical landmarks and lung cancer lesions, we collected both patients diagnosed with lung cancer and patients diagnosed with non-lung cancer who underwent biopsy via flexible bronchoscopy. As a result, the flexible bronchoscopy videos of 106 lung-cancer and 102 non-lung cancer patients were selected for detailed annotations.

### Video frame selection

For each patient in this study, the flexible bronchoscopy video was systematically converted into DICOM images at one-second intervals utilizing the *opencv* library. The patient’s identifiable personal information had been removed before the images were uploaded to an annotation system run on our private server. Senior bronchoscopists with extensive experience in this field chose qualified flexible bronchoscopy images, including anatomical landmarks and/or airway lesions. At least two additional physicians annotated and reviewed these images as part of a rigorous annotation process.

Generally, the selected images must meet the following criterias:

- Resolution: minimum resolution of 480 × 480 pixels.
- Light mode: standard white light, no special modes.
- Quality: no excessive darkness, blurriness, or shakiness.
- Content: clear display of anatomical landmarks and airway lesions.

### Data annotation

Referring to established bronchoscopy labels from clinical atlas^13,14^, bounding boxes and respective labels of objects related to anatomical landmarks and airway lesions were independently annotated on each image by two bronchoscopists with at least five years of experience. Subsequently, an expert with a minimum of 10 years of experience conducted a thorough review to finalize the annotations.

- For anatomical landmarks, we identified 11 common classes, including vocal cord, trachea, right main bronchus, left main bronchus, right superior lobar bronchus, intermediate bronchus, right middle lobar bronchus, right inferior lobar bronchus, left superior lobar bronchus, and left inferior lobar bronchus. For each image, anatomical landmark segments were precisely delineated with respect to their labels.
- For airway lesions, we chose typical lesions as described in published libraries^5,6^ of bronchoscopy images for annotation, including mucosal erythema, mucosal infiltration, tumor, mucosal edema of the carina, airway stenosis, anthracosis, and vascular growth. During the segmentation process, bronchoscopists were tasked with identifying and localizing each lesion according to the boundary of the lesion with surrounding areas.

Tables 1, 2 show the statistics of labels associated with the anatomical landmarks and lung cancer lesions, respectively.

**Table 1:** The statistics of anatomical landmarks.

#	Name	#patients lung cancer	#patients non-lung cancer	#images lung cancer	#images non-lung cancer
1	Vocal cords	60	72	120	149
2	Main carina	86	92	163	191
3	Intermediate bronchus	54	68	98	111
4	Right superior lobar bronchus	62	86	117	202
5	Right inferior lobar bronchus	71	85	125	200
6	Right middle lobar bronchus	71	84	134	187
7	Left inferior lobar bronchus	77	88	171	224
8	Left superior lobar bronchus	72	77	146	144
9	Right main bronchus	84	91	175	199
10	Left main bronchus	89	93	199	246
11	Trachea	36	49	50	75

**Table 2:** The statistics of lung cancer lesions.

#	Name	#patients lung cancer	#patients non-lung cancer	#objects lung cancer	#objects non-lung cancer
1	Mucosal erythema	16	19	35	42
2	Anthracosis	6	9	13	40
3	Stenosis	43	6	111	8
4	Mucosal edema of carina	42	9	128	28
5	Mucosal infiltration	74	5	355	9
6	Vascular growth	39	2	111	2
7	Tumor	40	0	183	0

### Data pre-processing

The annotated images were exported along with the corresponding metadata information, including labels and annotated segments in the json format. In the scope of our research, we aim to address two fundamental tasks, i.e., image segmentation and classification, for both anatomical landmarks and lesion detection.

**Fig. 2:**
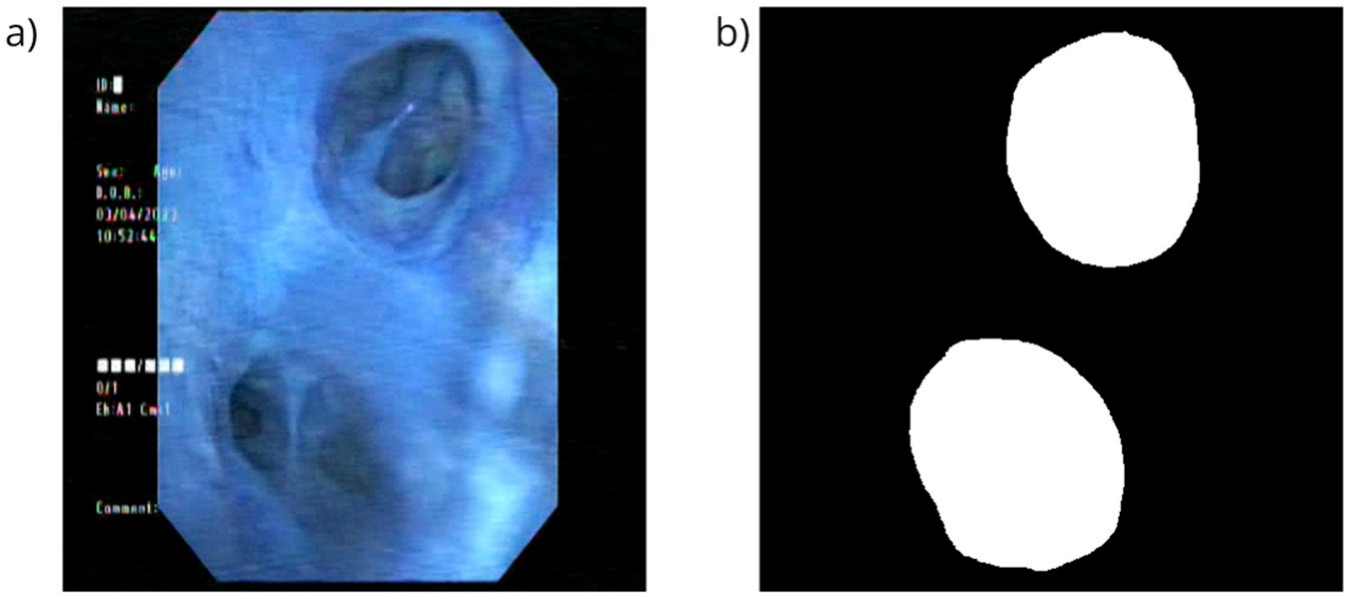
Binary mask transformation from (**a**) The original image to get (**b**) The transformed image.

**Fig. 3:**
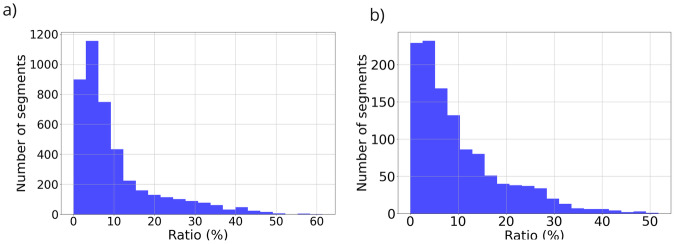
The histogram of the ratio between the annotated segment size and the image size on BM-BronchoLC for (**a**) Anatomical Landmark Segmentation and (**b**) Lesion Segmentation.

To extract the image segmentation, we created a mask for each raw input image as Fig. 2. The *objects.json* data file links patient ID, video ID and image ID. The *anotation.json* file consists of the object identifier for each specific polygon and its corresponding image. The *labels.json* file maps each object to a list of labels. We utilized *anotation.json* and *labels.json* to create the segmentation mask for each input image. For annotated labels of anatomical landmarks and lung cancer lesion segmentation, the output mask is a single channel image with the same dimensions as the input image. A value of 0 denotes a no-label pixel, while a value greater than 0 signifies a pixel belonging to a specific label type. To assist the dataset users, we have included a utility script *annots_to_mask.py* within the codebase to convert polygon annotations to binary masks. Figure 3 illustrates the histogram depicting the ratio (%) between the annotated segment size and the image size on BM-BronchoLC dataset. Notably, most segments were relatively small, representing small objects. This characteristic poses a significant challenge for the segmentation task.

For image classification, we need to align between an annotated segment and the respective label for every distinct object in the images. A new json file created for each object contains the information about the object id, label id and label name. Referring to the binary mask extracted from the annotation file, each object was defined by a tuple of (*object_id, masks, label*). We also separate objects from anatomical landmarks and lesions, respectively. Finally, we construct a unified JSON file by merging all object-level JSON files. We excluded all labels occurring less than 20 times within the dataset due to the insufficient statistics for effective learning. To facilitate the learning and evaluation of AI models, we partitioned the data into training, validation, and test subsets, as illustrated in Table 3.

**Table 3:** The statistics of training/validation/testing subsets for learning subtasks.

Types of Labels	#Images Train	#Images Valid	#Images Test
Anatomical Landmarks	1,549	173	192
Lung cancer lesions	574	64	71
Total	2,123	237	263

## Data Records

The BM-BronchoLC is accessible for download from the figshare repository^15^. We provide annotation files in json format, which are compatible with standard json viewer tools. The images within the dataset were stored with Portable Network Graphics (PNG) format which are compatible with standard image viewers.

The BM-BronchoLC dataset^15^ was organized into two primary folders, each representing a distinct patient category: lung cancer and non-lung cancer. These folders were compressed into *Lung_cancer.zip* and *Non_lung_cancer.zip* files. Each folder consists of the raw images extracted from patient videos and the associated metadata as described in the workflow depicted in Fig. 1. For the lung cancer category, the *imgs* folder contains the raw images and is stored following a specific path structure <*patient_id>/<video_id>/<image_id>.png*. These identifiers are anonymized strings and unique across the entire dataset. The three metadata files, i.e., *annotation.json*, *labels.json* and *objects.json* of the lung cancer data folder are included together with the images. The structure of the non-lung cancer folder mirrors that of the lung cancer folder.

## Technical Validation

For the technical validation, we seek to rigorously assess the dataset’s proficiency on two fundamental tasks, namely segmentation and classification. The segmentation assessment includes two subtasks, namely segmentation of anatomical landmarks and segmentation of lung cancer lesions. Similarly, for classification, we conducted two subtasks, namely classification of anatomical landmarks and lung cancer lesions. As a technical exploration effort, we will investigate two learning paradigms: single-task learning - tackles segmentation or classification separately, and multi-task learning - resolves the two tasks concurrently.

### Quality benchmarking on state-of-the-art methods

**Fig. 4:**
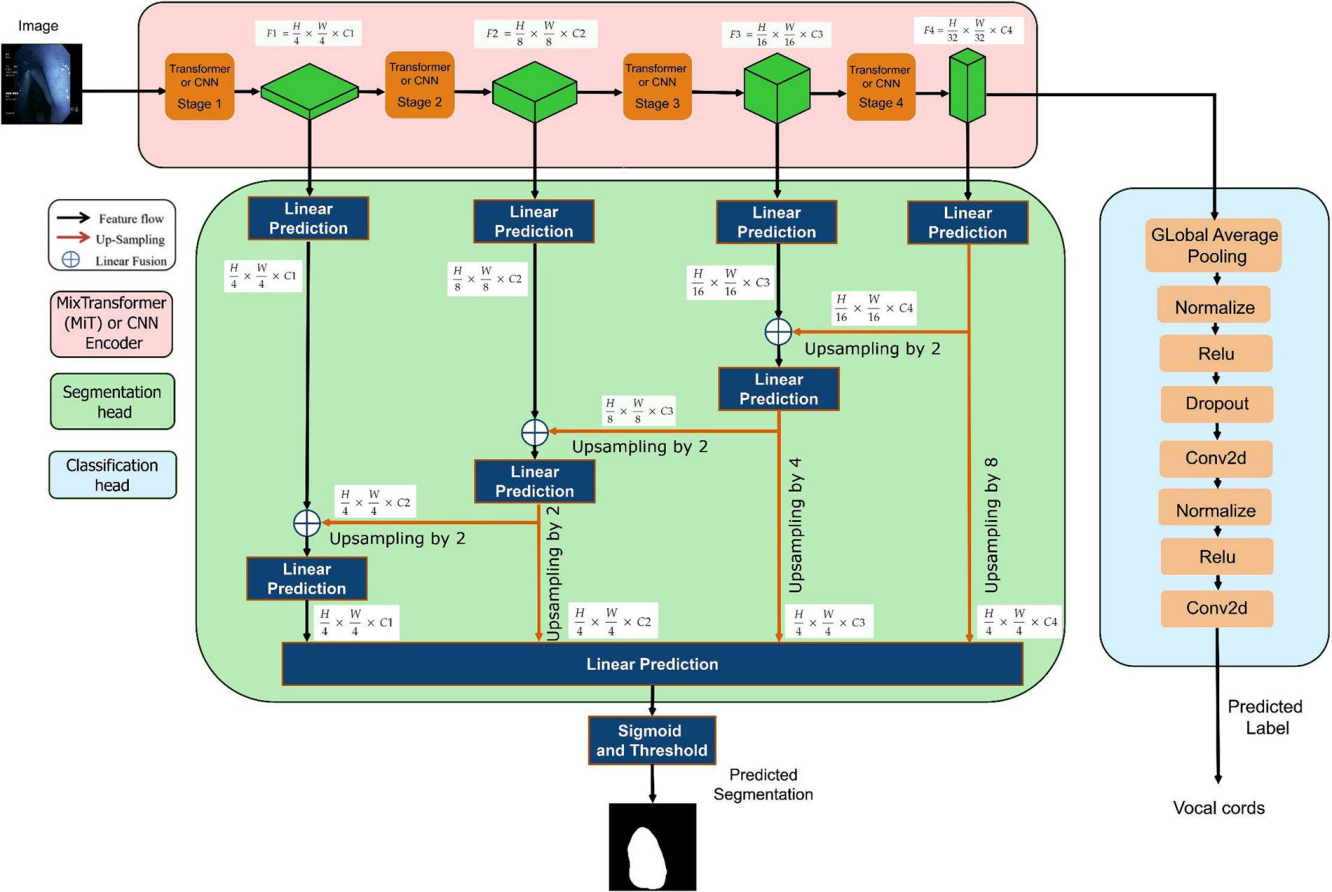
The overall architecture of the multi-task framework.

Figure 4 demonstrates the overall architecture of our benchmarking framework. This framework allows segmentation and classification components to be flexibly integrated or run independently. For segmentation, we have opted to focus on two typical backbone models namely Convolution Neural Network (UNet++) and Transformer (ESPFNet).

UNet++^16,17^ is an extension of the UNet^18^ architecture, which is a popular convolutional neural network (CNN) architecture for semantic segmentation tasks, particularly in the field of medical image analysis. UNet++ builds upon the UNet architecture, which consists of an encoder-decoder structure. The encoder extracts features from the input image through a sequence of convolutional and pooling layers. Meanwhile, the decoder upsamples the extracted features to generate a segmentation mask that aligns with the spatial dimensions as the original input image.

ESFPNet^19^ is a method to analyse fluorescence bronchoscopy videos in lung cancer diagnosis. This method employs a Mix Transformer (MiT) encoder as the backbone, coupled with an efficient phased feature pyramid (ESFP) as its decoder to generate the segmented output. The MiT encoder takes advantage of the Vision Transformers (ViT) network, incorporating four overlapping path fusion modules, each equipped with self-attention prediction in four stages. These stages provide both high-resolution raw- and low-resolution detailed features.

The joint model utilizes the Cerberus architecture^20^, a complete convolutional neural network with a shared encoder and an independent decoder making predictions for each task. Using a model as its backbone (ESPFNet or UNet++) in the encoder, Cerberus ensures a common representation is learned and that each task can leverage features learned by other tasks.

- Segmentation task: We employed a U-Net style decoder with incremental features by a factor of 2. Each resampling operation combines the features from the encoder with skip connections, followed by two convolution layers with 3 × 3 kernel and batch normalization.
- Classification task: we implement global average pooling to reduce features at the encoder output to a *k*-dimensional vector followed by two fully connected layers.

During the training phase, we employed seven NVIDIA GeForce RTX 2080 Ti GPUs. The data is divided into distinct training/validation/testing sets as outlined in Table 3. The specifics of the configuration on parameters such as learning rates, batch sizes, number of epochs, optimizers for each approach are presented in Table 4.

**Table 4:** The experimental settings.

#	Setting	ESPFnet	UNET++	ESFPNet (Multi-task)	UNet++(Multi-task)
1	Learning rate	0.0001	0.0001	0.0001	0.0001
2	Epoch	500	500	500	500
3	Batch size	8	4	8	4
4	Params	61.69 M	47.17 M	61.82 M	47.31 M
5	Init trainsize	352	352	352	352
6	Optimizer	AdamW	AdamW	AdamW	AdamW

### Evaluation metrics

#### Mean accuracy (MA)

It is utilized to evaluate the multi-label classification problem via *MA* = 1/*N* ∗ *A*_*i*_, where *N* represents the total number of classes and *A*_*i*_ is the accuracy for the *i*^*th*^ class, computed as the ratio of correctly predicted instances to the total number of instances for that label.

#### Dice coefficient (Dice)

It is used to validate the segmentation efficacy by *Dice* = (2*|*A* ∩ *B*|)/(|*A*| + |*B*|), where |*A* ∩ *B*| denotes the size (in terms of number of pixels) of the intersection between the predicted binary mask *A* and the ground truth binary mask *B*; |*A*|, |*B*| are as the size of the predicted- and ground-truth binary mask respectively.

### Experimental results

**Fig. 5:**
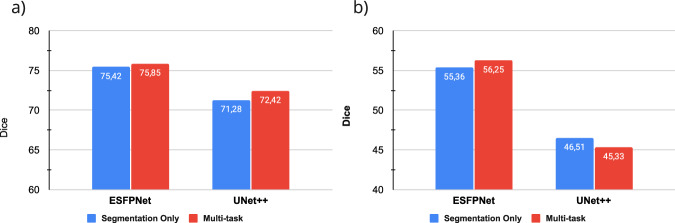
The model performance for the segmentation task on BM-BronchoLC namely: (**a**) Anatomical Landmark Segmentation, (**b**) Lesion Segmentation.

**Fig. 6:**
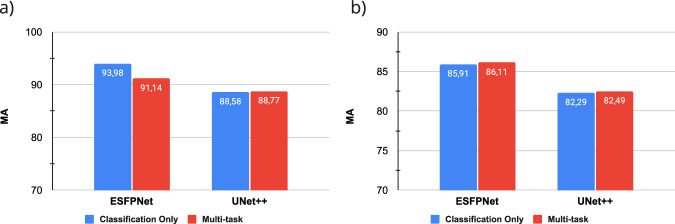
The model performance for the classification task on BM-BronchoLC namely: (**a**) Anatomical Landmark Classification, (**b**) Lesion Classification.

Figure 5 shows the comparative performance of the two backbone models, i.e., ESFPNet and UNet++, for the segmentation task. Notably, with the support of Transformer, the ESFPNet generally performs better than UNet++ across both single and multitask settings. Both models achieve a Dice coefficient of over 70% in segmenting anatomical landmarks. However, their effectiveness in the lesion segmentation is merely around 50%. The reason could be either the size of segment objects or the complex patterns of lung cancer lesions. For the classification task, we have similar observations via Fig. 6, in which ESFPNet outperforms UNet++ in various settings. All models have reasonable performance, ranging from 82 to 94% on the testing set, which validates the potential use of BM-BronchoLC.

**Fig. 7:**
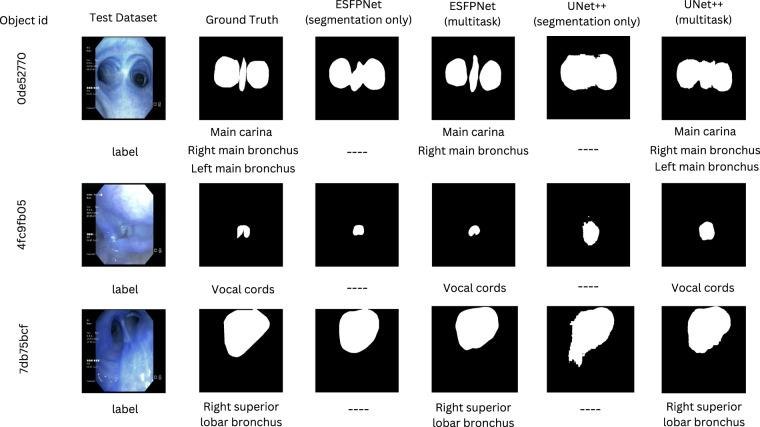
Quantitative comparison between multi-task versus single-task models for the anatomical landmark analysis.

**Fig. 8:**
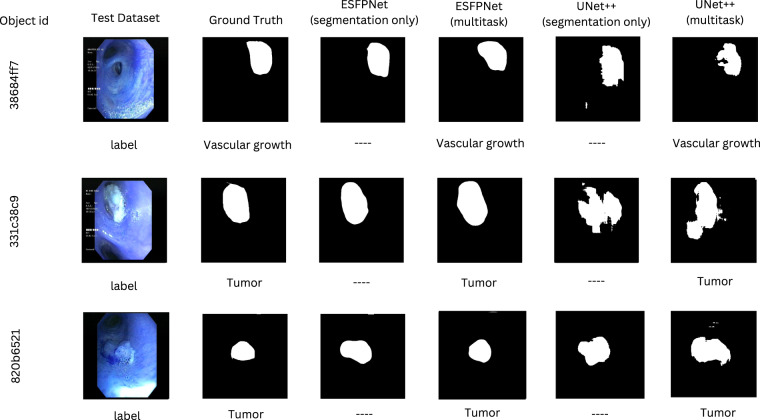
Quantitative comparison between multi-task versus single-task models for the lesion analysis.

Figures 7, 8 illustrate qualitative insights on how the two backbone models perform when predicting the segmentation and labels for the anatomical landmark and lung cancer lesion localization with single-task and multi-task settings. These visualizations align with the quantitative results, where the ESFPNet model generates smooth-and-accurate segments as well as precise labels in comparison to the UNet++ model.

## Data Availability

We hosted our codebase on the github repository: https://github.com/csuet/bronchoscopy_nsd. The code can be used to extract the segmentation and classification labels. It can also be used to train baseline models for single-task learning or multi-task learning. Please follow the instructions in the README.md file for further processing.
